# Terminal Protection of Small Molecule-Linked DNA for Small Molecule–Protein Interaction Assays

**DOI:** 10.3390/ijms15045221

**Published:** 2014-03-25

**Authors:** Cui Hu, Zhan Wu, Hao Tang, Li-Juan Tang, Ru-Qin Yu, Jian-Hui Jiang

**Affiliations:** State Key Laboratory of Chemo/Bio-Sensing and Chemometrics, College of Chemistry and Chemical Engineering, Hunan University, Changsha 410082, China; E-Mails: S12111016@hnu.edu.cn (C.H.); zhanwu@hnu.edu.cn (Z.W.); haotang@hnu.edu.cn (H.T.); rqyu@hnu.edu.cn (R.-Q.Y.)

**Keywords:** protein-ligand interaction, terminal protection, small molecule-linked DNA, signal amplification, nuclease

## Abstract

Methods for the detection of specific interactions between diverse proteins and various small-molecule ligands are of significant importance in understanding the mechanisms of many critical physiological processes of organisms. The techniques also represent a major avenue to drug screening, molecular diagnostics, and public safety monitoring. Terminal protection assay of small molecule-linked DNA is a demonstrated novel methodology which has exhibited great potential for the development of simple, sensitive, specific and high-throughput methods for the detection of small molecule–protein interactions. Herein, we review the basic principle of terminal protection assay, the development of associated methods, and the signal amplification strategies adopted for performance improving in small molecule–protein interaction assay.

## 1. Introduction

The affinity binding of small molecules with their target proteins relies on noncovalent but specific interactions, and small molecules that interact with proteins in this way serve as the affinity ligands of the associated proteins [1,2]. Specific interactions between proteins and small-molecule ligands are fundamental to the regulations of most physiological processes of organisms [3,4]. Small molecule–protein interaction assays, thus, are critical for revealing the mechanisms of many important physiological processes. Besides, the interaction assay techniques also represent a major avenue to drug screening, biomarker analysis in clinic, and public safety monitoring [5–8].

Some classic techniques, including affinity chromatography [9,10], kinetic capillary electrophoresis [11,12], fluorescence polarization [13,14] and surface plasmon resonance [15,16], have been developed for the detection of small molecule–protein interactions. However, problems such as the complex fixation of proteins or small molecules, limited sensitivity, potential nonspecific adsorption, or the requirement of sophisticated instruments frequently limit their widespread application. Beyond the aforementioned methods, Jiang and colleagues proposed a completely different concept of terminal protection assay for the investigation of small molecule–protein interactions [17]. They found an interesting phenomenon: the binding of a protein to a small molecule moiety at one terminus of a DNA module could protect the DNA from digestion by nucleases. Based on this finding, Jiang *et al.* as well as researchers from other groups have developed a series of methods for sensitive and specific detection of the interactions between proteins and small molecules [17]. The advantage of using terminal protection assay is that it translates the binding of small molecules to proteins into the presence of a specific DNA sequence, therefore enabling the detection of small molecule–protein interaction using various DNA sequence amplification and detection technologies [18–20].

This review traces the principles of terminal protection assay of small molecule-linked DNA and their applications in small molecule–protein interaction assay. In addition, some methods that share the concept of DNA protection assay are also discussed.

## 2. Terminal Protection Assay of Small Molecule-Linked DNA

**Figure 1.**
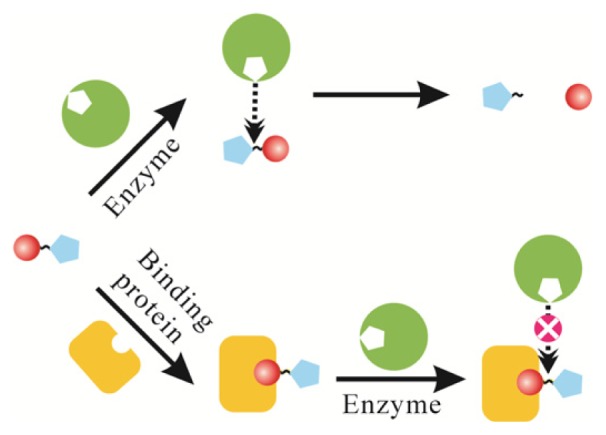
Stereo-hindrance effect caused by molecule recognition.

The primary hypothesis of terminal protection of small molecule-linked DNA was based on common biological effects of molecular recognition that biomacromolecules can have dramatic steric hindrance and thus inhibit the reactive activity at its binding site due to its large size. As illustrated in Figure 1, binding of a protein to a small molecule ligand may inhibit enzymatic reactions near the ligand because steric hindrance prevents an enzyme approaching the reaction site. Specifically, Jiang and colleagues found a general phenomenon that small molecule-linked DNA was still reactive with its exonucleases despite the introduction of a small molecule at the nucleotide of the DNA, but the substrate activity of DNA would be inhibited if there was significant steric hindrance around the reaction site of exonucleases, thus protecting the DNA from digestion [17]. Based on this finding, they designed two terminal protection strategies for single-stranded DNA (ssDNA) and double-stranded DNA (dsDNA), respectively.

### 2.1. Terminal Protection of Small Molecule-Linked Single-Stranded DNA

**Figure 2.**
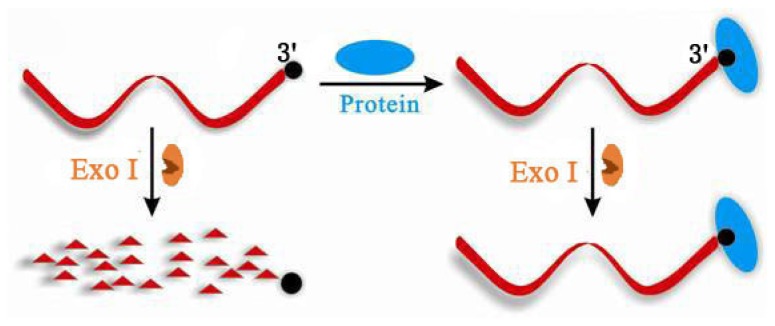
Working principle of terminal protection assay of small molecule-linked ssDNA.

Jiang *et al.* first investigated the behavior of exonuclease in reacting with small molecule-linked ssDNA in terminal protection assay [17]. Exonucleases are one kind of nuclease cleaving nucleotides from either the 3′ or 5′ end of a polynucleotide chain into mononucleotides, and exonuclease I (Exo I) can selectively degrade ssDNA to deoxyribonucleosides in a 3′–5′ direction. They found that after modifying a small molecule at the 3′ end of the ssDNA sequence, for example, a folate molecule on the terminal nucleotide at 3′ end, the ssDNA was still active for Exo I and can be hydrolyzed by this nuclease, as shown in Figure 2. However, with the binding of protein, as the steric hindrance at the 3′ termini of the ssDNA was dramatically increased, Exo I was prevented from accessing its reacting site and the hydrolysis reaction was inhibited. That is, the protein–small molecule interaction event protected the small molecule-labeled ssDNA from digestion by Exo I.

For a sensitive small molecule–protein interaction assay, Jiang *et al.* [17] proposed an electrochemical strategy by wrapping ligand-labeled DNA on single-walled carbon nanotubes (SWNTs) and the gold electrode was modified with a dense self-assembled monolayer (SAM) of 16-mercaptohexadecanoic acid (MHA) which isolated the electrode from the solution, blocking the electron transfer between redox solutes and resulting in no electrochemical signal. When Exo I was added into the reaction system, the folate-labeled DNA was hydrolyzed from its 3′ end and naked SWNTs were left, which could be isolated from the solution and assembled on the hydrophobic SAM. Due to the mediating electron transfer effect of SWNTs between the electrode and the electroactive substance, ferrocenecarboxylic acid, an enhanced redox current signal was observed. In the presence of target protein, folate receptor (FR), the interaction between FR and folate-labeled DNA prevented the Exo I-catalyzed hydrolysis reaction, thus leaving intact ssDNA on the surface of the SWNTs. As a result, no significant electrochemical signal was obtained. The proposed terminal protection assay strategy was demonstrated to be very sensitive in protein–small molecule interaction detection due to the natural electrochemical effect of SWNTs. A linear correlation in the concentration range from 10 pM to 1.0 nM of FR and a detection limit of 3 pM was achieved. Such a low detection limit was desirable for clinical applications. This strategy offeres a novel versatile platform for small molecule–protein interaction assays and a new means for rapid isolation of synthetic small-molecule ligands from libraries of small molecule-linked ssDNA.

Another novel electrochemical strategy based on the small molecule–linked DNA for interaction between small molecule and protein via a solid-state Ag/AgCl process, was developed by Chai and coworkers [21]. Biotin-labeled DNA was captured on the gold electrode and the binding of streptavidin (SA) prevented the DNA from being degraded by Exo I. Then the positively charged AuNPs was absorbed on the negatively charged DNA, which in turn catalyzed the silver deposited on AuNPs. The silver on AuNPs could be detected through a sensitive Ag/AgCl transformation process, producing an amplified electrochemical signal. In the absence of target protein SA, the biotin-labeled DNA was digested by Exo I and the AuNPs could not be absorbed, resulting in an obvious signal change. This method offered a detection limit as low as 10 pM in biotin–SA binding assays. The high sensitivity was attributed to the silver enhancement catalyzed by AuNPs and solid-state Ag/AgCl detection mechanism.

In addition to the electrochemical strategies on the basis of terminal protection assay, a graphene oxide-based fluorescent biosensor was also constructed for small molecule–protein interaction detection by Pang and his colleagues [22]. In this method, they designed a fluorophore-labeled ssDNA, which can adsorb on the surface of graphene oxide (GO) with fluorescence quenching. Another small molecule-labeled ssDNA that was complementary to the fluorophore-labeled ssDNA, was first incubated with the target protein followed by the addition of Exo I. Since the small molecule–protein interaction can inhibit the hydrolysis reaction of Exo I, intact small molecule-labeled ssDNA was left in the reaction solution and can be hybridized with the fluorophore-labeled ssDNA. Because the adsorbtion efficiency of dsDNA by GO was extremely low, the fluorescence was reserved and quantitatively indicated the small molecule–protein interaction event. Based on the fluorescence quenching and ssDNA adsorbing properties of GO as well as terminal protection assay principle, Su *et al.* [23] developed a similar strategy successfully used for small molecule–protein interaction assay. The two strategies both adopted graphene oxide as an energy receptor in resonance energy transfer, and made use of it to perform signal transduction.

### 2.2. Terminal Protection of Small Molecule-Linked Double-Stranded DNA

**Figure 3.**
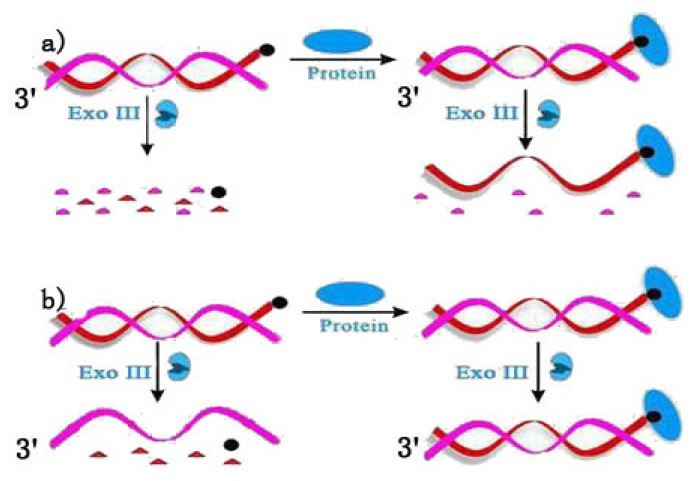
Working principles of terminal protection assay of small molecule-linked dsDNA (**a**) with 3′ end blunt or recessed; (**b**) with 3′ end overhang.

Terminal protection assay strategies were also constructed using small molecule-linked dsDNA. There are diverse exonucleases that could act on dsDNA, such as exonuclease III (Exo III), lambda exonuclease (Exo λ) and so on. The preferred DNA substrates of Exo III are blunt or recessed 3′ termini. Exo III is not active on ssDNA, and 3′ protruding termini are resistant to cleavage. Based on these properties, Figure 3 depicts two strategies of terminal protection of dsDNA for small molecule–protein interaction detection using Exo III. In Figure 3a, the dsDNA is designed to be blunt at its two 3′ termini with a small molecule labeled at the 3′ termini of one strand. In the absence of target protein, the two 3′ termini of dsDNA were both reactive to Exo III, so after the hydrolysis reaction, both strands of the DNA are digested. In contrast, the small molecule–protein interaction event inactivates the small molecule-labeled strand for Exo III, thus preventing this strand from digestion. When the 3′ termini of the unlabeled strand is overhanging as shown in Figure 3b, Exo III digests only the small molecule-labeled strand and reserves the unlabeled strand. On the other hand, protein binding events could protect the intact dsDNA from digestion by Exo III.

These strategies translate the detection of small molecule–protein interaction into probing a dsDNA or ssDNA. For signal transduction, Jiang *et al.* [24] designed a hairpin structure DNA with a small molecule labeled at its 3′ termini. In small molecule–protein interaction assay, the small molecule binding protein precluded the hydrolysis of the hairpin DNA against Exo III, resulting in an intact hairpin which was then stained by SYBR Green I. The small molecule–protein interaction events, thus, could be quantitatively explored by the fluorescence intensity of the dsDNA stain [24].

## 3. Signal Amplification in Terminal Protection Assay for Sensitive Detection of Small Molecule–Protein Interaction

**Figure 4.**
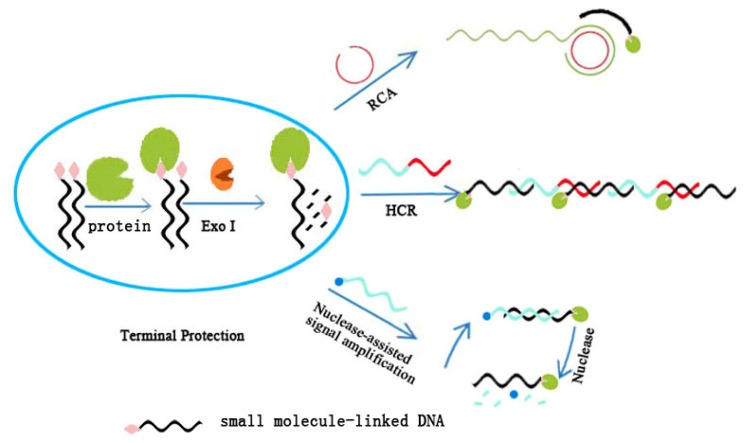
Signal amplification strategies adopted for terminal protection-based small molecule–protein interaction assay.

The success in finding terminal protection greatly contributes to the detection of small molecule–protein interaction. One of the greatest advantages of terminal protection assay is the permission of flexible signal amplification while using DNA as a basic component, which furnishes high sensitivity in small molecule–protein interaction assays [25,26]. Consequently, a variety of nucleic acid signal amplification techniques could be adopted in terminal protection assay for the detection of small molecule–protein interaction. The following sections review some signal amplification techniques that have been combined with terminal protection assay for the detection of small molecule–protein interactions (Figure 4).

### 3.1. Rolling Circle Amplification

**Figure 5.**
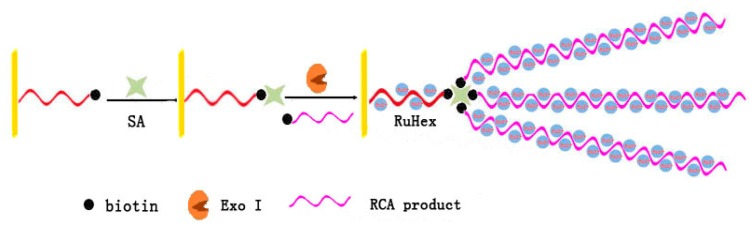
Schematic representation of the RCA integrated strategy for sensitive SA detection based on terminal protection of small molecule-linked DNA probes [28].

Rolling circle amplification (RCA) is an isothermal nucleic acid amplification strategy which forms a long ssDNA containing thousands of repeated sequence complementary to the circular template [27]. Combined with RCA, Chai *et al.* [28] have reported the development of an ultrasensitive electrochemical sensing method for biotin–SA interaction assay as illustrated in Figure 5. Two biotin-labeled ssDNA were designed. The short biotin-labeled ssDNA was self-assembled on an Au electrode, which would be digested by Exo I in the absence of the target protein, SA. Another biotin-labeled ssDNA was the ligation probe of RCA and prolonged in advance, which formed a biotin-labeled long-stranded RCA product. Due to its multiplex binding sites, SA bound with the fixed short biotin-labeled ssDNA and the biotin-labeled RCA product near the electrode at the same time. As a result, the long tail of the ssDNA adsorbed a large number of electroactive reporters, hexaamminerethenium (III) chloride (RuHex) via electrostatic interactions to give a highly amplified electrochemical signal. By incorporating the signal amplification technique of RCA, the strategy achieved a detection limit as low as 0.4 pM of SA with high selectivity.

### 3.2. Hybridization Chain Reaction

Hybridization chain reaction (HCR) accomplishes signal amplification via generating a long dsDNA with hundreds of repeated units in a series of hybridization events [29]. Wang *et al.* demonstrated an electrochemical method for the detection of small molecule–protein interactions by combining terminal protection assay with HCR [30]. In their assays, binding of FR to folate-labeled DNA precluded the degradation of DNA by Exo I. The protected DNA could then be captured by the DNA probe fixed on an electrode, and expose its free part for another ferrocene-labeled DNA probe. This ferrocene-labeled probe further hybridized with another protected DNA through its second region, and then its remaining part formed a G-quadruplex horseradish peroxidase (HRP)-mimicking DNAzyme at one end. The alternant hybridization between folate-labeled DNA and ferrocene-labeled DNA probes formed a supersandwich structure of DNA, which yielded abundant ferrocene and DNAzyme units on the surface of the electrode. Therefore, the detection of FR could be readily achieved using the redox current signal of the electrochemical catalyzed reduction of H_2_O_2_ in the presence of ferrocene and hemin/DNAzyme. The advantage of this strategy was that the signal amplification could be accomplished in a single-step, thus affording the method improved simplicity and sensitivity in small molecule–protein interaction assay.

### 3.3. Nuclease-Assisted Signal Amplification

#### 3.3.1. Nickase-Assisted Signal Amplification

Nucleases, both of endonucleases and exonucleases, are useful tools in developing DNA signal amplification methods. Nickase is a kind of endonuclease which needs a specific recognition site in dsDNA but only cleaves one strand of the duplex [31]. If there are enough ssDNA to form new duplex with the uncleaved strand, nickase allows recycling cleavage of DNA duplex. In terminal protection assay, nickase-assisted signal amplification can be readily achieved by introducing a recognition site in the sequence of small molecule-labeled DNA. A sensitive electrochemical method based on nickase-assisted signal amplification has been developed by Li *et al.* for the detection of FR–folate interaction [32]. In this method, binding FR to the folate-labeled ssDNA prevented the ssDNA from digestion by Exo I. The intact folate-labeled ssDNA then hybridized with the ssDNA immobilized on the surface of electrode to form a nickase site. It thus initiated a cycle of nickase cleavage, ssDNA release and DNA hybridization. Recycling cleavage of the ssDNA immobilized on the electrode weakened the blocking effect against [Fe(CN)_6_]^3−/4−^, accordingly resulting in an increased electrochemical signal. The method was demonstrated to have a detection range of 0.3–20 ng/mL for FR and can be used for the investigation of small molecule–protein pairs with nanomolar dissociation constants.

#### 3.3.2. Exo III-Assisted Signal Amplification

In Zhou’s study, they developed a strategy of Exo III-assisted recycling cleavage of fluorescent probe for SA–biotin interaction detection [33]. Based on Exo III-assisted DNA cleavage, they achieved “turn off” and “turn on” strategies simultaneously. In the “turn off” strategy, small molecule biotin was labeled at the 3′-terminus of the antisense strand of the trigger strand that was involved in the cycle of signal amplification and the trigger strand was the 3′ end overhang. When there was no streptavidin, the antisense strand was degraded stepwise from the 3′ to 5′ termini by Exo III, releasing the trigger strand which subsequently hybridized with a molecule beacon (MB) to recover the fluorescence. The formed duplexes of the trigger strand and the opened MB were further degraded by Exo III from the 3′ blunt end of the MB to release the trigger strand and open a new MB, thus rendering a signal generation cycle. In contrast, binding of SA to the biotin-labeled DNA inhibited the degradation of the antisense strand and no trigger strand was released to initiate the signal generation cycle. In the “turn on” strategy, the biotin was attached to the 3′ end of the trigger strand and the two strands were completely complementary to each other. In this case, the two strands could be both digested by Exo III and no signal generated. On the contrary, in the presence of streptavidin, only the antisense strand can be degraded by Exo III. Then the released biotin-labeled trigger strand opened the MB, thus rendering a signal generation cycle. The signal amplification strategy furnished the streptavidin assay an improved low detection limit of 0.8 fM.

### 3.4. Dual Amplification Strategy Based on Rolling Cycle and Exo III-Assisted Recycling Cleavage

**Figure 6.**
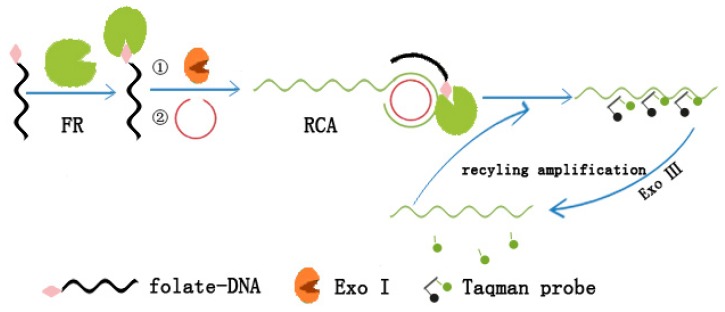
Schematic representation of the dual amplification based on terminal protection assay [34].

Combining the RCA technique with an exonuclease-assisted recycling cleavage of fluorescent probe in terminal protection assay, Chu *et al.* [34] have realized a dual amplification strategy for small molecule–protein interaction detection (Figure 6). In this method, small molecule-labeled ssDNA was protected from digestion by Exo I via binding to its target protein and initiated a RCA process. Then, the RCA product was probed by fluorescence-quenched probes (taqman probes) at its repeated sequences. The hybrid of the RCA product with the taqman probes, in turn, could be hydrolyzed by Exo III to separate the fluorophore from its quencher. With the hydrolysis of the taqman probes, the RCA product was re-exposed to intact taqman probes. As a result, the Exo III-assisted recycling cleavage of the taqman probes released abundant fluorescent reporters. Due to the advantage of the dual signal amplification, the method may have a great potential for the detection of small molecule–protein pairs with low affinities.

## 4. Other Methods

### 4.1. Non-Nuclease-Assisted Terminal Protection Assay

The terminal protection assay strategies proposed by Jiang and his group utilized the unique properties of exonucleases. Without the participation of exonuclease, Wu *et al.* constructed a small molecule-linked DNA conversion for screening the small molecule–protein interaction [35]. This non-nuclease-assisted terminal protection assay relied on small molecule–protein binding events-mediated protection of a small molecule-labeled DNA duplex from being immobilized onto a gold substrate. In this method, the DNA duplex was a hybrid of a 3′ thiolated ssDNA and an ssDNA with a small molecule label at 5′ end. Because of its intrinsic self-assembly behavior, the thiolated DNA is easy to be immobilized onto the surface of a gold substrate. With the interaction between the protein and small molecule, the steric hindrance dramatically increased around the small-molecule labeling site which was adjacent to the thiolated site of the other strand. Thereby, it inhibited the self-assembly of the DNA duplex on the gold substrate. Based on such a strategy, Wu *et al.* [35] have accomplished the quantitative detection of interaction between β-indole acetic acid (IAA) and its antibody on a platform of quartz-crystal-microbalance (QCM).

### 4.2. DNA/Fok I Transducer

**Figure 7.**
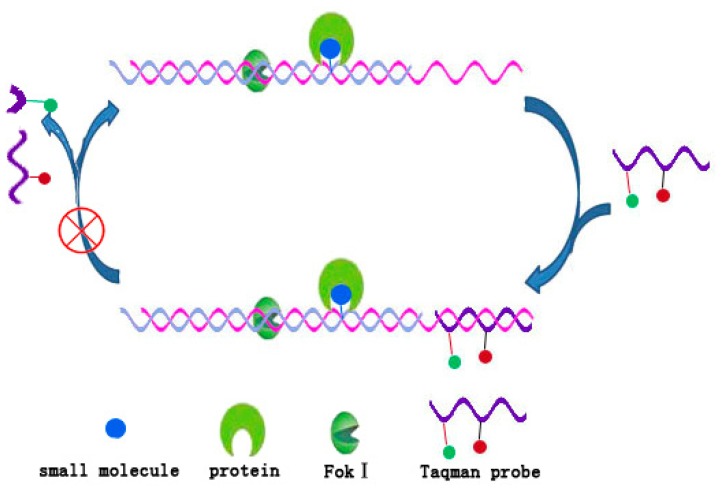
Working principle of DNA/Fok I transducer for small molecule–protein interaction assay [36].

The foundation of terminal protection assay of DNA is that binding a protein to small molecule-labeled DNA will dramatically increase the steric hindrance around the binding site and thus inhibit the action of exonucleases. According to the principle of altering work environment of enzymes, Jiang *et al.* [36] proposed a more generalized concept of the protection assay of DNA and have constructed a DNA/Fok I transducer as a sensitive platform for detection of small molecule–protein interaction [36]. Fok I is a kind of endonuclease which recognizes the sequence 5′-GGATG-3′ of duplex DNA and cleaves the ninth and thirteenth nucleotide located on the downstream of the recognition site [37]. Like other nucleases, Fok I is also highly sensitive to the circumstance around the recognition site. This makes it possible to produce differential signal before and after the protein binding events. As shown in Figure 7, a small molecule-labeled heteroduplex DNA and Fok I constructed a DNA/Fok I transducer. In the absence of target protein, the small molecule label was not large enough to influence the Fok I activity and the transducer cyclically cleaved the taqman probe, which continued to activate fluorescence. On the contrary, in the presence of target protein, the steric hindrance caused by small molecule–protein interaction inhibited the Fok I activity and no DNA probe could be cleaved. Thereby, only very weak fluorescence signal was observed. In this study, Jiang *et al.* [36]have demonstrated that the DNA/Fok I transducer could be used to detect the interactions with dissociation constants ranging from subnanomoles to micromoles and had the potential to become a universal, sensitive and selective platform for quantitative assays of small molecule–protein interactions [36].

## 5. Conclusions

The review traces the recent development in the field of small molecule–protein interaction assays upon the terminal protection of small molecule-labeled DNA. Terminal protection is a generalized discovery demonstrated by Jiang *et al.* [17], that small molecule–DNA chimeras could be protected from degradation by various DNA exonucleases. Since terminal protection converts small molecule–protein interaction assays into the detection of DNA of various structures, diverse DNA sequence amplification and detection technologies may be utilized. Combining varying DNA amplification techniques, such as RCA, HCR, or DNA nuclease-assistant recycling amplification, subsequently improves the sensitivity of small molecule–protein interaction assays. Moreover, different signal readout approaches of DNA detection allow the development of highly specific, simple, cost-efficient, rapid, robust and high-throughput methods for small molecule–protein interaction assays. To sum up, terminal protection assay of small molecule-linked DNA serves as a versatile tool for interrogating the interaction between protein and small molecule ligands. With the pursuit of simple, high-throughput and highly sensitive analytical methods, terminal protection assay is expected to hold considerable potential in small molecule–protein interaction investigation and related studies.
